# Rapidly Evolving Acute Brainstem Syndrome With Respiratory Insufficiency due to Intracranial Dural Arteriovenous Fistula

**DOI:** 10.1155/crnm/8666719

**Published:** 2026-05-04

**Authors:** Hong Kiat Lim, Duncan Street, Wen-Xern Chong, Amrit-Deep Samra

**Affiliations:** ^1^ Department of Neurology, York Hospital, York and Scarborough Teaching Hospitals NHS Foundation Trust, Wigginton Road Clifton, York, YO31 8HE, UK, yorkhospital.com; ^2^ Department of Neurology, University Hospitals Coventry and Warwickshire NHS Trust, Clifford Bridge Road, Coventry, CV2 2DX, UK, nhs.uk; ^3^ Department of Radiology, University Hospitals Coventry and Warwickshire NHS Trust, Clifford Bridge Road, Coventry, CV2 2DX, UK, nhs.uk

## Abstract

A 52‐year‐old female presented with one week of progressive quadriparesis followed by the development of diplopia, dysphonia and dysphagia over 48 h. Initial impression was of an inflammatory myelitis given mild cerebrospinal fluid pleocytosis and diffuse cervical cord swelling extending to the lower brainstem on MRI. Treatment with intravenous methylprednisolone was commenced. Her condition continued to progress with a brief cardiac arrest secondary to diaphragmatic insufficiency due to brainstem involvement. She survived the cardiac arrest after a period of cardiopulmonary resuscitation and was admitted to the intensive care unit. Subsequent MRI suggested a chronic right sigmoid venous sinus thrombosis with venous congestion in the brainstem and upper cervical cord. Digital subtraction angiogram revealed a right dural arteriovenous fistula (DAVF) between the right transverse venous sinus and perimesecephalic veins. The dural arteriovenous fistula was embolised with subsequent rapid improvement in bulbar function. Limb weakness also improved, and she was transferred to a rehabilitation unit for physiotherapy. Our case highlights the importance of rapid recognition of an intracranial DAVF mimicking an inflammatory myelitis in the setting of an acute brainstem syndrome. Symptoms can progress quickly if untreated with the risk of progression following the administration of corticosteroids.

## 1. Introduction

Intracranial dural arteriovenous fistulae (DAVFs) are abnormal connections between dural venous sinuses or cortical veins and dural arteries without an intervening capillary formation [1]. Their development can be associated with venous sinus thrombosis (VST); however, whether the VST is the cause or consequence of DAVFs remains a subject of debate. Clinical features at presentation are varied and depend on the fistula location, degree of arteriovenous shunting and pattern of venous drainage [2].

We present a case of DAVF associated with a VST causing multiple cranial neuropathies, myelopathy and cardiorespiratory compromise.

Our case is unique and interesting due to the combination of a DAVF and VST. Whilst cases of intracranial DAVF and/or VST have been reported in literature, we have not come across a presentation as fulminant as seen in our case.

## 2. Case Presentation

A 52‐year‐old female with well‐controlled hypertension and asthma presented to the emergency department at a district hospital with a seven‐day history of worsening quadriparesis. Prior to presentation, she was fully mobile and independent with activities of daily living. She denied any previous head injury.

Initial assessment revealed asymmetric quadriparesis (left worse than right) with normal deep tendon reflexes and upgoing plantar response bilaterally. Initial MRI head showed increased signal on T2 and Fluid‐Attenuated Inversion Recovery (FLAIR) sequences in the medulla and cervical spinal cord (see Figures 1(a) and 1(c)). Lumbar puncture on the day of admission showed an opening pressure (OP) of 29 cm H_2_O. Cerebrospinal fluid (CSF) protein was 1.11 g/L and CSF glucose 3.5 mmol/L. CSF white cell count (WCC) was 10 × 10^6^/L (60% polymorphs). CSF culture and viral PCR were negative. Results of CSF oligoclonal bands (OCBs) were subsequently received and were negative. At a later stage, results of serum aquaporin 4, myelin oligodendrocyte glycoprotein (MOG) and glial fibrillary acidic protein (GFAP) antibodies were received, and these were negative.

FIGURE 1Magnetic resonance imaging pre‐ and postembolization. (a) Midcoronal T2 FLAIR image demonstrating profound oedema of the medulla and upper cervical cord. (b) Midcoronal T2 FLAIR image demonstrating resolution of oedema postembolization. (c) Axial T2 image demonstrating venous infarct with swelling of the dorsal left medulla (double arrows) and partial preservation of the grey‐white matter differentiation on the right (double arrowhead). Right sigmoid sinus thrombosis is also observed (single arrow) with black “flow void” signal representing patent left sigmoid sinus (single arrowhead). (d) Axial T2 image demonstrating resolution of medullar oedema postembolization.

**Figure:**
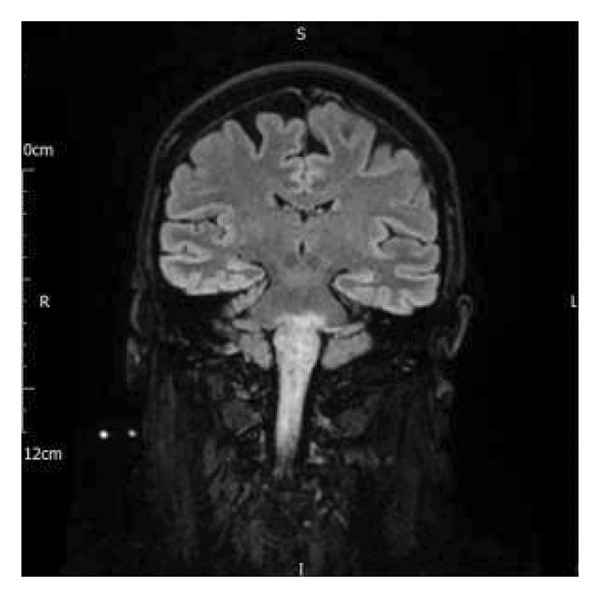
(a)

**Figure:**
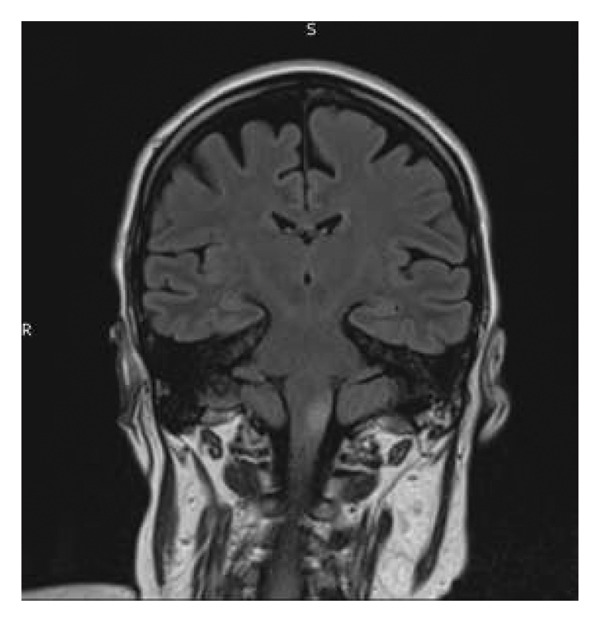
(b)

**Figure:**
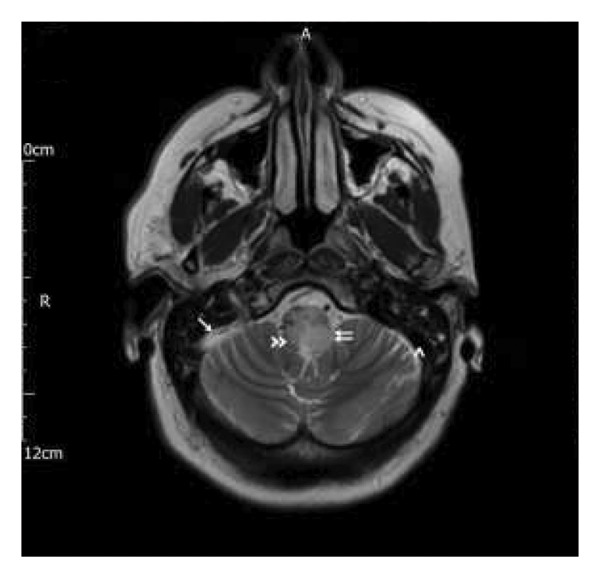
(c)

**Figure:**
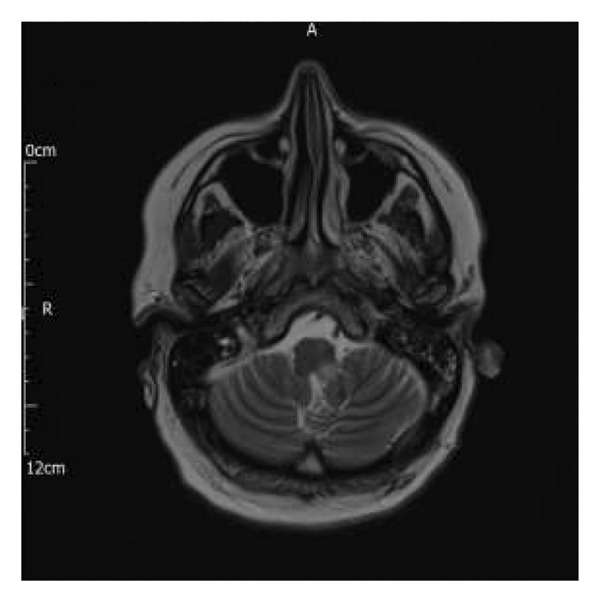
(d)

A second lumbar puncture was done 3 days after the first. It showed WCC 8 × 10^6^/L (100% polymorphs), CSF protein 0.44 g/L and CSF glucose 5.6 mmol/L. CSF OCBs were again negative.

Twenty‐four hours later, she was transferred to the tertiary neurosciences centre and on admission, features of marked dysphonia, dysphagia and sustained down beating nystagmus on left lateral gaze were evident. There was marked quadriparesis, worse in lower limbs, and ataxia in her left upper limb. MRC power grading was 4‐/5 in both upper limbs, 2 in right leg and 3 in the left leg. Soon after her transfer and over the course of a couple of hours, her breathing became more shallow and rapid. She suffered a respiratory arrest, was intubated and admitted to the intensive care unit (ICU). She received 5 days of intravenous methylprednisolone and 2 sessions of plasma exchange due to the initial suspicion of an inflammatory myelitis such as neuromyelitis optica spectrum disorder (NMOSD).

Her imaging was subsequently reviewed by a neuroradiologist specialising in vascular imaging. There was a suspicion of a right sigmoid VST given abnormal flow void on T2 sequences (see Figure 1(c)). Furthermore, the brainstem oedema was associated with a degree of venous congestion as the left perimesecephalic veins were prominent on the left side of the medulla. A small acute infarct was also seen in the left lateral medulla.

She was weaned off ventilation after 5 days and stepped down to the ward. At nadir, there was complete quadriplegia and complete palatal palsy. Appearances on a subsequent MRI venogram and angiogram strengthened the suspicion of a right sigmoid VST.

She suffered a cardiac arrest the day after stepdown, with an approximate downtime of 10 min and an underlying rhythm of ventricular tachycardia. She was intubated and readmitted to ICU.

**FIGURE 2:**
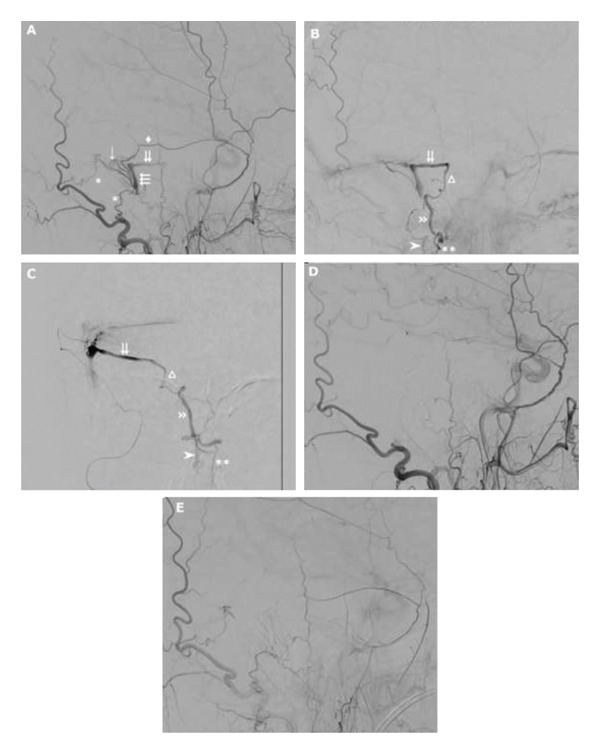
Digital subtraction angiography pre‐ and postembolization. (A) Lateral view from external carotid artery approach preembolization in the arterial phase (asterisk = occipital arteries, single arrow = dura transition, triple arrows = dural arteriovenous fistula vein, diamond = middle meningeal artery, double arrows = right superior petrosal sinus). (B) Lateral view from external carotid artery approach preembolization in the venous phase demonstrating venous reflux along peri mesencephalic venous system (double arrows = right superior petrosal sinus, triangle = lateral anterior pontine mesencephalic vein, double arrowhead = lateral mesencephalic vein, single arrowhead = posterior spinal vein, double asterisk = anterior spinal vein). (C) Anterior–posterior view from temporal branch of right middle meningeal artery using microcatheter demonstrating venous reflux from right superior petrosal sinus down peri mesencephalic venous system to cervical cord (double arrows = right superior petrosal sinus, triangle = lateral anterior pontine mesencephalic vein, double arrowhead = lateral mesencephalic vein, single arrowhead = posterior spinal vein, double asterisk = anterior spinal vein). (D, E) Lateral view from external carotid artery approach postembolization in early (D) and late (E) arterial phase demonstrating successful embolization of dural arteriovenous fistula and obliteration of dural arteriovenous fistula vein.

Cerebral angiography showed a right‐sided DAVF fed by the right middle meningeal and right occipital artery with branches into the right sigmoid sinus and draining into the venous system surrounding the medulla oblongata (see Figure 2(A)). Chronic thrombosis of the right transverse and sigmoid sinuses with new collateral venous drainage was also noted. Successful Onyx embolization of skull‐based DAVF was performed, and therapeutic anticoagulation (enoxaparin) was started following haematology advice.

Over the next few days, there was sustained improvement in her bulbar function and limb weakness. Repeat MRI head showed significant improvement of brainstem and upper cervical spinal cord swelling (see Figures 1(b) and 1(d)). There were features of an established left medullary infarct. She was transferred to a neurorehabilitation unit for therapy. Bulbar dysfunction had fully resolved by discharge to the neurorehabilitation unit. She was started on a direct oral anticoagulant (dabigatran), following haematology advice. The sequence of events during her admission is summarised in Table 1.

**TABLE 1:** Sequence of events during the admission.

Day	Event
1	Admission to district hospital
2	First MRI scan
3	First Lumbar puncture: OP 29 cm H_2_O, CSF protein 1.11 g/L, CSF glucose 3.5 mmol/L, WCC 10 × 10^6^/L (60% polymorphs). CSF culture, viral PCR, OCB negative.
4	Transfer to tertiary neurosciences centre. Started on IV methylprednisolone. Respiratory arrest and admission to ITU
6	Second Lumbar puncture: CSF protein 0.44 g/L, CSF glucose 5.6 mmol/L, WCC 8 × 10^6^/L (100% polymorphs). CSF OCBs were negative.
7	Review of MRI images with neuroradiologist
8	Stepdown to the ward. IV methylprednisolone stopped after completing 5‐day course. MRI angiogram and venogram performed
9	Cardiac arrest. Intubated and readmitted to ICU
10	Cerebral angiography and embolization of DAVF
11	Started on treatment with therapeutic anticoagulation (enoxaparin)
30	Stepdown to the ward. Started physiotherapy.
37	Repeat MRI with improved appearances.
42	Transfer to the neurorehabilitation unit.

She was last reviewed in clinic approximately 15 months postdischarge. She was able to walk up and down stairs under supervision. She uses a walking frame to mobilize and a wheelchair for longer distances. She was able to push herself out of the wheelchair on her own. Modified Rankin Scale (mRS) score was 3. Outpatient haematology review found no obvious cause for the VST. Given her clinical improvement and low risk of recurrence of VST, dabigatran was stopped after 16 months. She has since started taking low‐dose aspirin (75 mg once daily).

In summary, the relatively normal cell counts on both CSF samples taken whilst the case was rapidly evolving raised doubt about the preliminary diagnosis of an inflammatory myelitis. Furthermore, the presence of a right venous sinus thrombosis, left medullary infarct and brainstem oedema with associated venous congestion was inconsistent with an inflammatory myelitis and was suspicious for a vascular aetiology such as an intracranial DAVF.

## 3. Discussion

Intracranial DAVFs can present with nonspecific symptoms and mimic other neurological conditions. The clinical presentation can include headaches, cranial nerve palsies, myelopathy and subarachnoid haemorrhage [1, 3, 4]. It is important to be aware of this rare condition as it can progress rapidly and lead to fatal consequences if not treated correctly. In our case, a DAVF and cerebral VST caused brainstem venous congestion, which led to rapidly progressive quadriplegia with bulbar and cardiorespiratory compromise.

Intracranial DAVFs tend to occur in middle‐aged adults, with median age in the sixth decade. Trauma, previous craniotomy, thrombosis and thrombophilia are thought to be predisposing factors [5]. DAVFs can be associated with VST, in which the thrombosis is thought to be secondary to venous hypertension from the fistula [2]. Conversely, VST can promote formation of DAVFs either by stimulating the opening of dormant AV shunts or by inducing the formation of AV shunts via neo angiogenesis [5]. It is not clear in our case whether the DAVF led to the chronic VST or vice versa.

In terms of diagnostic imaging, noncontrast CT is often low yield. MRI head with T2 sequencing can show features of DAVFs, such as abnormal flow voids which should prompt further imaging [6]. Magnetic resonance angiography and CT angiography can show the DAVF itself, feeding arteries and draining veins. Catheter‐based cerebral angiography is, however, the gold standard to provide the diagnosis [1].

Treatment of DAVFs depends on clinical presentation and incurs the risk of serious complications. DAVFs with brainstem venous congestion and myelopathy, such as in this case, warrant endovascular treatment given the high risk of mortality [5]. Endovascular treatment is the current mainstay in obliterating the underlying DAVF. Surgery can be considered in cases where endovascular treatment has failed [3]. Those without high‐risk features can be managed conservatively with clinical and radiological follow‐up [5]. With appropriate identification and treatment, outcomes (as measured by mRS) are good [2].

Administration of intravenous corticosteroids in cases of untreated spinal arteriovenous fistulas can, however, lead to worsening of symptoms. A retrospective review was conducted by Nasr et al. on patients with spinal DAVF who received corticosteroids. Of the 13 patients who received IV methylprednisolone, seven patients (53.8%) reported worsening of symptoms and did not make a full neurological recovery [7]. Rain et al. reported neurological symptoms worsening following administration of intravenous corticosteroids for a case of presumed transverse myelitis. A spinal dural AV fistula was discovered on repeat spinal cord imaging [8]. Whittam et al. reported a case series involving 6 patients with intracranial DAVF causing brainstem or cervical cord oedema. The initial presentations varied in severity, ranging from progressive walking difficulty to progressive mild tetraparesis. Four out of 5 patients who received corticosteroids for presumed inflammatory myelitis suffered an acute deterioration [9].

The cause for this phenomenon is not clear although two explanations have been proposed. The first is related to the mechanism of action of corticosteroids. In addition to suppressing inflammation, corticosteroids promote fluid and sodium retention in blood vessels through mineralocorticoid activity. Such activity is thought to increase venous engorgement and impede venous outflow from the DAVF, consequently worsening spinal cord oedema. The second is through a direct venous thrombogenic effect. Venous hypertension generated through thrombosis eliminates the pressure gradient within the capillaries leading to reduced gaseous exchange and medullary ischaemia. This medullary oedema and ischaemia can precipitate cardiorespiratory compromise [7, 8, 10].

Inflammatory myelitis, for which steroids are the mainstay of treatment, is nevertheless an important differential diagnosis to consider in cases with a rapidly involving brainstem syndrome. In the early stages of presentation, it is often difficult to distinguish due to a lack of a clear and simple biomarker, leading to potentially detrimental use of steroids or other immunomodulatory agents. Chen et al. reported a case of acute paraparesis with bulbar involvement which was treated initially with immunomodulatory medications for presumed brainstem encephalitis and cervical myelitis. A cranial DAVF was later found on cerebral angiography [1]. Koutsiompa et al. reported a case involving a subacute progressive myelopathy with MRI of the cervical spine which was suggestive of a DAVF. The initial cervical angiography was normal, but an infratentorial DAVF was later identified on a repeat angiogram. This was treated by craniotomy and clipping of the DAVF [11]. Chawla et al. described a recent case with a 2‐month history of asymmetric progressive quadriparesis with bulbar symptoms mimicking cervicomedullary myelitis which deteriorated following the administration of intravenous methylprednisolone and intravenous immunoglobulin. A posterior fossa DAVF was later found on catheter angiogram and treated with embolization with significant improvement in symptoms [12]. We propose close liaison with neuroradiology in the early stages of unexplained brainstem or spinal cord swelling to temper early corticosteroid use, thus lowering the potential risk of neurological deterioration.

## Author Contributions

Dr Hong Kiat Lim wrote the manuscript. Dr Wen‐Xern Chong, Dr Duncan Street and Dr Amrit‐Deep Samra reviewed and revised the manuscript.

## Funding

No funding was received for this research.

## Ethics Statement

Written informed consent has been obtained from the patient for submission of case report manuscript for publication.

## Conflicts of Interest

The authors declare no conflicts of interest.

## Data Availability

The data that support the findings of this study are available from the corresponding author upon reasonable request.
